# Abolished adherence alters signaling pathways in phorbol ester-induced human U937 cells

**DOI:** 10.1186/1478-811X-9-20

**Published:** 2011-09-22

**Authors:** Anna Otte, Katharina Mandel, Gesche Reinstrom, Ralf Hass

**Affiliations:** 1 Biochemistry and Tumor Biology Lab, Gynecology Research Unit, Dept. of Gynecology and Obstetrics, Medical University, Hannover, Germany

## Abstract

Phorbol ester (TPA) treatment of human U937 myeloid leukemia cells is associated with increasing adherence and monocyte-like maturation whereby the role of β_2 _integrin-mediated attachment for subsequent growth properties and the differentiation program remains unclear. Here, stably-transfected U937 cells with a pMTH1 vector containing the β_2 _integrin gene of CD11b in antisense orientation (asCD11b-U937) demonstrated a significantly reduced proliferative capacity in contrast to control vector transfectants (pMTH1-U937) or wild-type U937 cells. Phorbol ester exposure induced adherence and growth arrest in more than 90% of pMTH1-U937 and wild-type U937 cells after 72 h. In contrast, TPA-treated asCD11b-U937 failed to attach and the proliferation continued in more than 30% of the cells. Moreover, increased apoptosis appeared in asCD11b-U937 after TPA induction in contrast to pMTH1-U937 cells. In addition, non-specific inhibition of adherence on an agarose surface demonstrated internucleosomal DNA fragmentation in both, pMTH1-U937 and asCD11b-U937 after TPA treatment indicating a functional relationship between abolished adherence, regulation of proliferation and induction of apoptosis. Western blot analysis revealed differences in the expression levels and altered phosphorylation patterns of Pyk-2, pp60src and p42/p44 MAP kinases between pMTH1-U937 and asCD11b-U937 following TPA exposure which was also substantiated by Pyk-2 immunoprecipitation. These findings suggested that induced adherence predominantly mediated by a functional CD11b/CD18 integrin in U937 cells is involved in the activation of downstream signaling kinases and contributes to cell cycle regulation and apoptosis during monocytic maturation.

## Introduction

Human myeloid leukemia cells represent an experimental model for a monocyte/macrophage-like maturation [1,2] and effects of differentiation-inducing agents on cell adherence and filament expression have been extensively characterized in previous studies [3]. Non-adherent and autonomously proliferating myeloid leukemia cells including the human U937 cell line can be induced to differentiate along the monocyte/macrophage pathway following stimulation with the phorbol ester derivate 12-O-tetradecanoyl-phorbol-13-acetate (TPA). Whereas TPA activates the Ca^2+ ^and phospholipid-dependent isoforms of protein kinase C, the course of TPA-induced macrophage-like differentiation in U937 cells is also accompanied by changes in the cAMP metabolism and cAMP is known to regulate important intracellular signaling processes [4]. Morphological changes of differentiating U937 cells are accompanied by cellular adherence and these effects are paralleled by an enhanced expression of the β_2 _integrins CD11a, CD11c, CD18, and particularly CD11b [5]. The CD11a, CD11b and CD11c glycoproteins represent the α-subunits of a heterodimeric association with the common β-subunit CD18, respectively, to build a functional β_2_ integrin which are involved in two different cellular mechanisms of adherence: 1) adhesion to solid surfaces such as plastic and 2) adhesion to adjacent cells. Thus, previous work has demonstrated that a polyphenol-mediated up-regulation of CD11b in certain T cell subsets resulted in an increased adherence to plastic, whereas a down-modulation of CD11b in monocytes was associated with significantly reduced attachment to the plastic surface [6]. For adhesion to adjacent cells during the formation of cell-to-cell contacts and intercellular communication processes, junctional adhesion molecules can associate through their extracellular domains with the CD11b/CD18 (CR3/Mac-1) β_2 _integrin contributing for example to the regulation of leukocyte-endothelial cell interactions [7].

Studies in a differentiation-defective subclone of the U937 cell line, termed TUR (TPA-U937-resistant), have demonstrated that this population fails to express significant levels of CD11b after TPA treatment [8]. The TUR have been characterized for a defect to relay a sufficient threshold of phosphorylation signals downstream of Raf-1 kinase whereby previous work emphasized the importance of Raf-1 kinase in the regulation of cellular proliferation and differentiation [9]. Whereas the kinase signalling defects in the TUR leukemia cells are likely to be associated with the proliferative capacity, confirmative studies in colorectal cancer cells have demonstrated that high Erk activity can effect cell growth by targeting cell cycle regulators such as p27^kip1 ^[10]. In association with the Raf-1 signaling defect, the human TUR leukemia cells are unable to attach and continue to proliferate in response to a phorbol ester stimulation [11] indicating a functional contribution of CD11b to these effects beyond the involvement in the regulation of cell attachment. Indeed, β_2 _integrin-mediated signalling pathways may also affect components involved in the extracellular matrix formation substantiating the importance of a tumor cell microenvironment [12].

Other studies have demonstrated that a down-modulation of the CD11b integrin fails to develop certain markers of a monocytic phenotype following exposure to the differentiation-inducing TPA [13]. However, little is known about the role of CD11b within the monocytic differentiation program and how CD11b integrin-mediated cell attachment may also affect restructure of the extracellular matrix and a possible involvement in the cell cycle progression and intracellular metabolic pathways which are also relevant for maturation along the monocytic lineage.

In the present study, it was of interest therefore, to examine the role of the CD11b integrin and cell adherence for metabolic changes and the proliferative capacity after phorbol ester treatment of these human leukemic cells.

## Material and methods

### Cell culture

Human U937 myeloid leukemia cells (American Type Culture Collection #CRL-1593.2) were cultured in RPMI 1640 containing 10% of heat-inactivated fetal bovine serum, 100 units/ml penicillin, 100 μg/ml streptomycin, and 2 mM L-glutamine in a 37°C humidified atmosphere with 5% CO_2_. Moreover, U937 cells stably transfected with the pMTH1 vector (pMTH1-U937) and U937 cells stably transfected with the pMTH1 vector containing the CD11b gene in antisense orientation (asCD11b-U937) [13] were cultured under similar conditions. The functionality of the CD11b down-modulation has been demonstrated by CD11b immunofluorescence staining [13]. The different cell populations were incubated with 5nM of the differentiation-inducing agent 12-O-tetradecanoylphorbol-13-acetate (TPA) (Sigma Chemie GmbH, Taufkirchen, Germany) for different time points as indicated.

### Cell cycle analysis

The cell cycle analysis and ViCell analysis was performed as described previously [14]. Briefly, 5 × 10^5 ^U937 cells, pMTH1-U937 control or asCD11b-U937 were exposed to 5 nM TPA for 24 h, 48 h and 72 h, respectively. Following incubation, the different populations were fixed in 70% (v/v) ice-cold ethanol at 4°C for 24 h. Thereafter, the fixed cells were stained with CyStain DNA 2 step kit (Partec GmbH, Münster, Germany) and filtered through a 50 μm filter. The samples were then analyzed in a Galaxy flow cytometer (Partec) using the MultiCycle cell cycle software (Phoenix Flow Systems Inc., San Diego, CA). The subG_1 _population which represents dying or dead cells was calculated as the rate of apoptotic cells.

### Apoptosis assay

Following an appropriate 24 h TPA treatment in the presence or absence of an agarose surface, 1 × 10^6 ^pMTH1-U937 control cells and asCD11b-U937 cells were harvested by centrifugation, washed with PBS and incubated in 20 μl of 50 mM Tris-HCL (pH 8.0), 10 mM EDTA, 0.5% SDS, and 0.5 μg/ml proteinase K (Sigma Chemical GmbH) for 1 h at 50°C, respectively. Thereafter, 10 μl of 0.5 μg/ml (DNase free) RNase was then added and the incubation continued for an additional hour. The digested samples were incubated with 10 μl of 10 mM EDTA (pH 8.0) containing 2% (w/v) low-melting-point agarose, 0.25% bromophenol blue, and 40% (w/v) sucrose at 70°C. The DNA was separated in a 2% agarose gel according to a DNA base pair (bp) length marker and visualized by UV illumination after ethidium bromide staining.

### Immunoblot analysis

Untreated and 5 nM TPA-stimulated pMTH1-U937 and asCD11b-U937 cells were washed three times in ice-cold PBS and lysed in a buffer containing 10 mM Tris-HCl (pH 7.6), 140 mM NaCl, 10 mM EDTA, 1% (v/v) NP-40 with the addition of 10 μg/ml aprotinin, 10 μg/ml leupeptin, and 1 mM phenylmethylsulfonylfluoride (PMSF) (all from Sigma). Protein concentration of the cell lysates was adjusted using the colorimetric BCA-assay (Perbio Science Deutschland, Bonn, Germany), subjected to SDS-polyacrylamide gel electrophoresis and transferred to a PVDF membrane (Millipore GmbH, Schwalbach, Germany). The membranes were blocked with PBS containing 5% FCS and 0.05% Tween-20 (PBS/Tween). After washing four times with PBS/Tween, the membranes were incubated with the primary antibodies (polyclonal anti-c-Jun (Geneka Biotechnology Inc., Montreal, Canada); monoclonal anti-p21^WAF-1/sdi-1^, clone SX118 (BD Biosciences, Heidelberg, Germany); monoclonal anti-vimentin, clone VIM 13.2 (Sigma Chemie GmbH, Taufkirchen, Germany); polyclonal anti-caspase-3, cleaved at Asp175 (Cell Signaling Technologies, Danvers, MA, USA) monoclonal anti-FAK, clone 77 (BD Biosciences, Heidelberg, Germany); polyclonal anti-Pyk-2 (Biomol GmbH, Hamburg, Germany); pp60src, polyclonal anti phopho pp60src (Tyr^416^) (NEB/Cell Signaling Technology, Frankfurt/Main, Germany), MAP kinase, monoclonal anti-phospho-p44/42 MAP kinase, clone E10 (NEB/Cell Signaling Technologies) and monoclonal anti-β-actin, clone C4/actin (BD Biosciences), for 2 h/37°C, washed four times with PBS/Tween and incubated with the appropriate horseradish peroxidase-conjugated secondary antibody (all from Santa Cruz Biotechnology, Santa Cruz, CA) for 1 h/37°C. The membranes were washed with PBS/Tween and visualized by autoradiography using the ECL-detection kit (GE Healthcare, München, Germany).

### Immunoprecipitation of Pyk-2

After swelling and washing of protein-A sepharose CL-4B (GE Healthcare, München, Germany) in PBS according to the manufacturer's instructions, the resuspended sepharose was coupled with an anti-Pyk-2 antibody (Santa Cruz Biotechnology Inc., Heidelberg, Germany) for 1 h at 4°C. In parallel, cell lysates from untreated and 5 nM TPA-stimulated pMTH1-U937 and asCD11b-U937 cells were adjusted to similar protein amounts according to the BCA-assay and pre-absorbed to uncoupled sepharose for 1 h at 4°C. Following centrifugation (14,000 g/5 min) and removal of the sepharose beads, the pre-absorbed cell lysates were incubated with the anti-Pyk-2-coupled sepharose for 2 h at 4°C. After 5× washing in RIPA-buffer, the sepharose beads carrying the immunoprecipitates were subjected to a SDS-polyacrylamide gel electrophoresis and transferred to a PVDF membrane (Millipore GmbH). Subsequent Western blotting was performed by using a polyclonal anti-Pyk-2 antibody (Biomol GmbH) and a monoclonal anti-phophotyrosine antibody, clone 4G10 (Upstate Inc., Lake Placid, NY, USA) and the blots were visualized by autoradiography using the ECL-detection kit (GE Healthcare).

## Results

**Figure 1:**
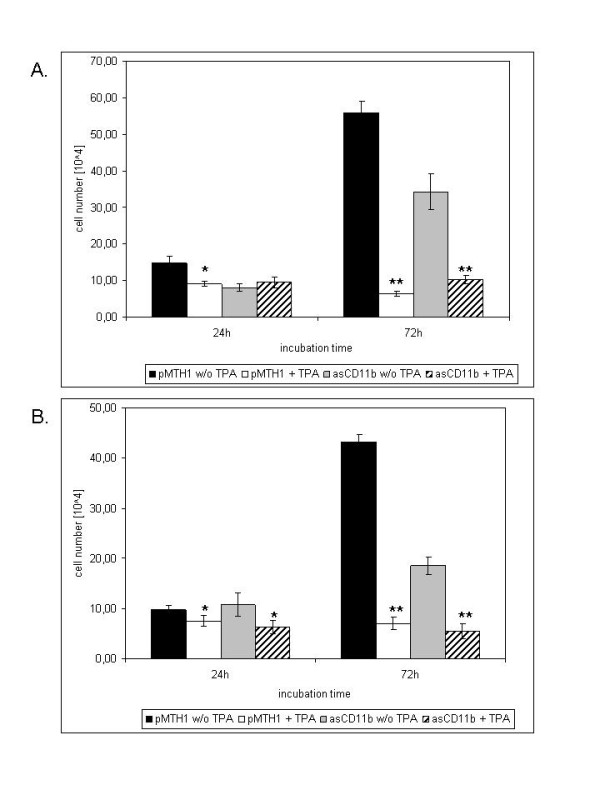
**Quantification of viable cells in TPA-treated pMTH1-U937 and asCD11b-U937 cultures**. **A**. The pMTH1-U937 and asCD11b-U937 cells were incubated in 24-well plates at a density of 5 × 10^4^ cells/ml in the absence or presence of 5 nM TPA for up to 72 h, respectively. The number of viable cells in the different populations was measured by trypan blue exclusion. Adherent cells were detached for cell counting by Trypsin/EDTA treatment. Data represent the mean ± s.d. of four independent experiments. Statistical significance was evaluated by paired student's t-test (p < 0.005 (*); p < 0.001 (**)) for the comparison of untreated and corresponding TPA-treated populations. **B**. The pMTH1-U937 and asCD11b-U937 cells were cultured at a density of 5 × 10^4^ cells/ml in 24-well plates precoated with 2% agarose to non-specifically block cellular adherence. Following incubation in the absence or presence of 5 nM TPA for up to 72 h, the number of viable cells in the different populations was measured by trypan blue exclusion. Data represent the mean ± s.d. of four independent experiments. Statistical significance was evaluated by paired student's t-test (p < 0.005 (*); p < 0.001 (**)) for the comparison of untreated and corresponding TPA-treated populations.

The proliferative capacity of pMTH1-U937 and asCD11b-U937 cells was measured after induction of differentiation with 5 nM TPA providing either plastic adherence in normal culture dishes (uncoated plates) or the culture onto a layer of 2% agarose to non-specifically block cell-to-substrate adherence (Figure 1). Thus, pMTH1-U937 cells demonstrated a continuously increasing proliferation whereby TPA treatment resulted in a significant growth arrest after 72 h (Figure, 1A). These proliferative and morphological findings were in absolute agreement with data obtained from wild-type U937 cells [5]. In contrast, asCD11b-U937 transfectants exhibited a significantly reduced steady-state proliferation as compared to pMTH1-U937 both, in uncoated and agarose-coated plates (Figure 1A, B). Moreover, incubation of asCD11b-U937 with 5 nM TPA transiently slowed the cell growth to about 30% of the cells in contrast to a marked growth inhibition of more than 90% of TPA-treated pMTH1-U937 within 72 h (Figure 1A). In addition, asCD11b-U937 population reduced the cell viability and showed little if any cell adhesion in either uncoated or agarose-coated plates following exposure to the phorbol ester (data not shown).

With respect to the morphology, proliferating pMTH1-U937 as single cells in normal culture dishes exhibited both, a cell-to-cell and a cell-to-matrix (e.g. plastic) adherence by the formation of firmly-attached three-dimensional cell aggregates after 72 h of TPA exposure which has been similarly documented in TPA-treated U937 cells [5]. Following culture of pMTH1-U937 on an agarose surface, the formation of three-dimensional cell aggregates could be observed again after phorbol ester incubation, however, these cell aggregates were floating in suspension and failed to attach to the matrix. Moreover, this TPA-induced pMTH1-U937 population on agarose-coated culture dishes demonstrated a variety of disintegrated and dead cells after 72 h (data not shown). Likewise, TPA-treated asCD11b-U937 transfectants revealed numerous disintegrated and dead cells after TPA exposure, however, this accumulation of cell death was already observed in normal culture dishes and did not significantly alter in the presence of agarose (data not shown).

**Figure 2:**
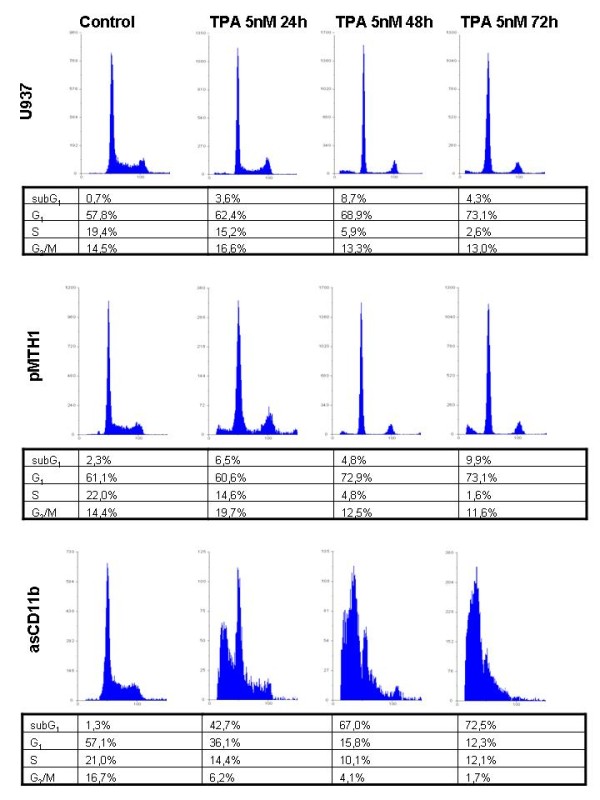
**Cell cycle analysis of U937, pMTH1-U937 and asCD11b-U937 cells**. U937 cells, pMTH1-U937 and asCD11b-U937 cells were incubated in 6-well plates at a density of 5 × 10^4 ^cells/ml in the absence or presence of 5 nM TPA for up to 72 h, respectively. At the time points indicated representative aliquots of each population were fixed in 70% ethanol, stained with a CyStain DNA kit and subjected to cell cycle analysis by flow cytometry. The percentage of cell cycle distribution was gated and calculated using the Multi-cycle software.

According to the altered morphology and proliferation in the different populations cell cycle analysis was performed to evaluate changes in the cell cycle distribution (Figure 2). Wild-type U937 and pMTH1-U937 cells showed similar distributions displaying a loss of S phase cells from about 20% down to about 2% and a significant accumulation in G_0_/G_1 _phase from about 58% up to more than 70% within 72 h of TPA treatment (Figure 2). In contrast, asCD11b cells continued to progress into S phase after TPA exposure which remained at about 12% (Figure 2). Moreover, a progressive accumulation of apoptotic subG_1 _cells became detectable in asCD11b cells ranging from less than 2% in the untreated population to more than 70% after 72 h of TPA exposure (Figure 2).

**Figure 3:**
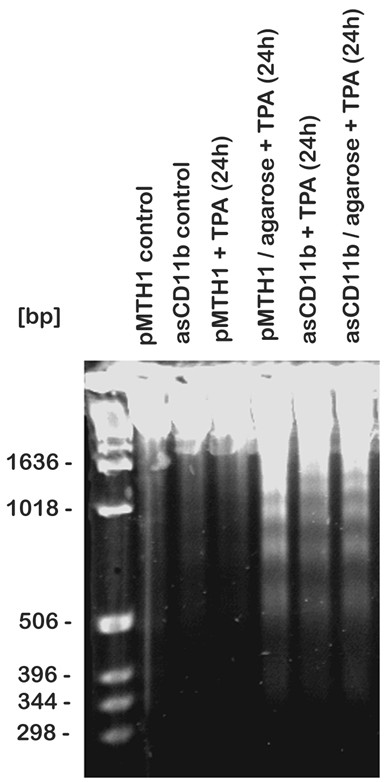
**Analysis of apoptosis by internucleosomal DNA fragmentation**. Untreated pMTH1-U937 (pMTH1 control) and untreated asCD11b-U937 (asCD11b control) were incubated with 5 nM TPA at a density of 5 × 10^4^ cells/ml either in uncoated or in 2% (w/v) agarose-precoated cell culture dishes for 24 h, respectively. Following DNA extraction the samples were analyzed in a 2% agarose gel and visualized by UV illumination after ethidium bromide staining. The cell cycle histograms were analyzed in the Flomax program (Partec) and the percentage of the distinct cell cycle phase has been calculated using the MultiCycle cell cycle software (Phoenix Flow Systems Inc., San Diego, CA).

These findings indicated that together with the failed attachment after TPA treatment, the asCD11b-U937 cells remain in the proliferative cell cycle which may lead to apoptosis. Indeed, analysis of internucleosomal DNA fragmentation as one marker for apoptosis revealed little if any detectable DNA fragments in untreated pMTH1-U937 and asCD11b-U937 cells, as well as in the 24 h TPA-exposed pMTH1-U937 population in uncoated culture dishes (Figure 3). However, in agarose-coated dishes, the 24 h TPA-treated pMTH1-U937 demonstrated significant DNA laddering according to the DNA base pair standard which was paralleled by 24 h TPA-treated asCD11b populations independent of uncoated or agarose-coated dishes (Figure 3). Together, these findings suggested that TPA-treated U937 cells allowed for cell attachment cease to divide and progress with a differentiation program. In contrast, TPA-treated U937 cells with an abolished cell attachment underwent apoptosis indicating that the adherence-associated β2 integrin CD11b may play a significant role in mediating signals for the regulation of proliferation and differentiation.

**Figure 4:**
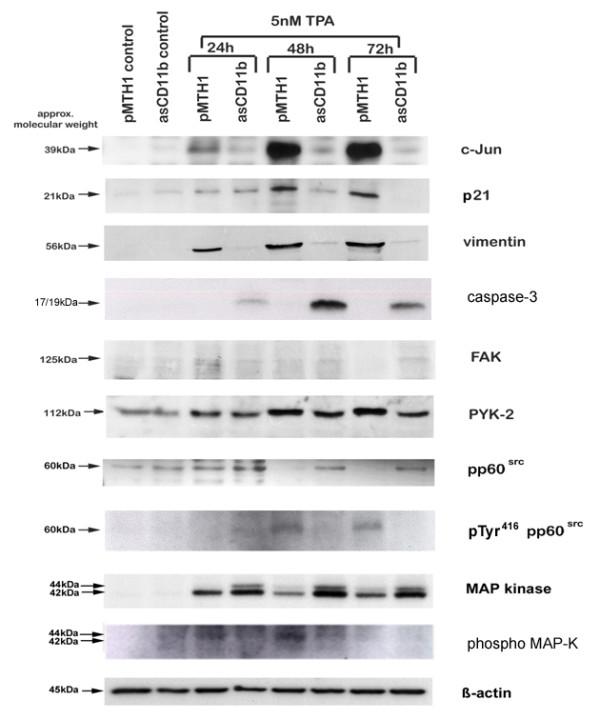
**Western blot analysis**. The pMTH1-U937 (pMTH1) and asCD11b-U937 (asCD11b) cells were cultured in the absence and in the presence of 5 nM TPA for 24 h up to 72 h, respectively. Thereafter, the different populations were harvested, lyzed and 40 μg of total cellular protein was. For the expression of growth and differentiation markers, antibodies against p21, c-Jun and vimentin were applied. Kinase expression was tested for FAK, PYK-2, pp60^src ^and MAP kinase, respectively. The unaltered expression level of β-actin serves as a loading control.

In order to further test the hypothesis of differentiation defects and functional alterations due to the down-modulation of the CD11b integrin, Western blot analysis was applied to differentiation markers, kinase expression and metabolic factors. Whereas TPA treatment of pMTH1-U937 cells was associated with a significant induction of the c-Jun protein within 72 h, little if any induction of this transcription factor was detectable in asCD11b-U937 cells (Figure 4). Likewise, the cell cycle inhibitor p21^sdi-1 ^and the differentiation marker vimentin were significantly expressed after TPA exposure of pMTH1-U937 cells in contrast to asCD11b-U937 cells (Figure 4). For the detection of apoptosis products, cleaved fragments (17/19 kDa) of caspase-3 were detectable exclusively in TPA-treated asCD11b-U937 cells with a significant increase between 24 h to 48 h (Figure 4).

**Figure 5:**
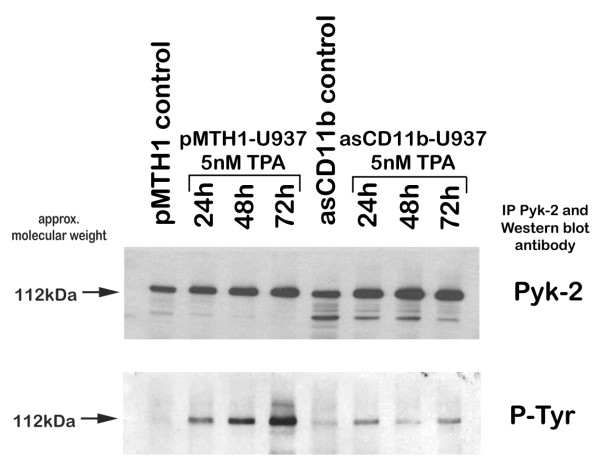
**Immunoprecipitation of Pyk-2**. The pMTH1-U937 (pMTH1) and asCD11b-U937 (asCD11b) cells were cultured in the absence and in the presence of 5 nM TPA for 24 h up to 72 h, respectively. Thereafter, the different populations were harvested, lyzed and after pre-absorbation incubated with sepharose-conjugated anti-Pyk-2. Precipitated Pyk-2 samples (IP Pyk-2) were then separated by 10% SDS-PAGE followed by Western blotting for Pyk-2 equal loading and for Pyk-2 phosphorylation by phosphotyrosine (P-Tyr).

Signaling compounds for β2 integrins involve p125 focal adhesion kinase (FAK) and the 112 kDa Pyk-2 kinase. Whereas little FAK expression was detectable in the different populations, Pyk-2 protein levels were slightly enhanced in pMTH1-U937 cells as compared to asCD11b-U937 cells following phorbol ester stimulation (Figure 4). Likewise, tyrosine phosphorylation of Pyk-2 as determined in immunoprecipitation experiments was progressively elevated in pMTH1-U937 cells in contrast to asCD11b-U937 after TPA exposure for 24 h to 72 h (Figure 5). Downstream signaling kinases including pp60^src ^and the p42/p44 MAP kinases revealed a more pronounced protein expression of these kinases in TPA-treated asCD11b-U937 rather than pMTH1-U937 cells (Figure 4). Conversely, with respect to the signalling pMTH1-U937 cells demonstrated an enhanced phosphorylation of p42/p44 MAP kinases and phopho pp60^src ^(P-Tyr^416^) between 24 h and 72 h after TPA exposure as compared to asCD11b-U937 cells (Figure 4).

## Discussion

Phorbol ester treatment of non-adherent human myeloid leukemia cells is associated with cell attachment to form 3-dimensional cell aggregates, growth arrest and monocytic differentiation, and conversely, the reversible process of retrodifferentiation and rejuvenation reveals the loss of previously acquired monocytic features, a regained proliferative capacity and cell detachment to form again a single cell suspension of monoblastoid precursor cells [3,15]. Whereas these findings suggested a tight regulatory relationship between adherence, cell growth and metabolic changes, previous work has documented a predominant role of the β_2 _integrin CD11b in TPA-induced adherence of human leukemic cells [5]. This function of CD11b appears even more pronounced by its involvement to affect the cell growth as indicated by the significantly reduced cell number after down-modulation of CD11b. TPA exposure further diminished the persisting growth activity in asCD11b cells, however, the amount of viable cells remained significantly higher after 72 h as compared to a complete growth arrest in control transfectants suggesting a functional role β_2 _integrins in the balance of adherence and the regulation of U937 cell growth. This hypothesis is also substantiated by the non-specific inhibition of adherence in the agarose-coated plates after 72 h of TPA treatment, whereby the amount of asCD11b cells remained at a similar level as compared to pMTH1 control transfectants which were unable to adhere to the agarose surface. Moreover, the steady-state proliferation on agarose without phorbol ester exposure already demonstrates differences in the proliferative capacity between pMTH1 control U937 cells and asCD11b transfectants suggesting regulatory effects by β_2 _integrins on the U937 cell growth independent of TPA-mediated differentiation signals.

Following TPA-induced adherence and subsequent differentiation pMTH1 control transfectants like wild-type U937 cells arrested predominantly in G_0_/G_1 _of the cell cycle and accordingly expressed the cell cycle inhibitor p21^sdi-1 ^in contrast to asCD11b-U937 cells displaying a progressive accumulation of a non-attached population in the subG_1 _phase with characteristics of apoptosis. The importance of attachment for subsequent differentiation in these cells is also substantiated in TPA-treated pMTH1 on agarose whereby the failure of matrix adherence resulted in apoptosis despite of a significant CD11b induction. Taken together, these findings suggest that a sufficient expression of β_2 _integrins in differentiating U937 cells represents a prerequisite for subsequent matrix adherence-mediated growth arrest and a functional monocytic maturation program. Conversely, down-modulation of β_2 _integrins abrogated the cell adherence and consequently, continuing proliferation signals can interfere with and derail the concomitant induction of factors associated with the monocytic phenotype eventually resulting in apoptosis as indicated by DNA fragmentation and caspase-3 cleavage.

A functional β_2 _integrin composition in monocytic cells is involved in adhesion, transendothelial migration and phagocytosis [16] and it can relay downstream signals for cell polarity via the small Rho GTPase Cdc42 in neutrophils [17], however, the detailed signalling mechanisms of the β_2 _integrins via downstream kinases to contribute to a regulatory balance between adhesion-mediated cell cycle progression and a paralleled differentiation program remain unclear.

Integrin signalling-mediated cellular activation predominantly involves focal adhesion kinase (FAK) and related adhesion focal tyrosine kinase (RAFTK), also known as cell adhesion kinase-ß (CAK-ß) or proline-rich tyrosine kinase (Pyk) 2 [18]. β2-integrin-mediated activation of FAK is associated with autophosphorylation and a transient downstream activation of SH2 adaptor proteins such as Grb2 and kinases including phosphatidylinositol-3-kinase and distinct src kinases. Pyk-2 can regulate cell spreading and migration after CD11b/CD18-controlled adhesion [19]. Whereas signalling cascades relayed by FAK, Pyk-2, pp60^src ^and MAP kinase are conferred via phosphorylation at Ser/Thr and/or Tyr residues, the present study indicated that the significantly reduced threshold of phosphorylation signals in phorbol ester-induced asCD11b-U937 as compared to the pMTH1-U937 control transfectants may be insufficient to relay stable downstream signals for growth arrest and differentiation. This hypothesis is further substantiated by the elevated protein levels of pp60^src ^and MAP kinase in asCD11b-U937 after TPA stimulation suggesting that the increased expression of downstream kinases cannot compensate the failure of a required upstream signal to relay an adequate threshold of phosphorylation signals.

Together, in addition to an altered phosphorylation pattern, inhibition of CD11b/CD18-mediated adherence also affects the expression levels of the kinases during TPA-induced differentiation suggesting much more profound alterations in the overall cell function and fate since phosphorylation signals can be adapted and changed much faster than a kinase protein expression profile.

Recent data in murine macrophages demonstrated a CD11b/CD18-dependent expression of pro-inflammatory cytokines via activation of FAK/PI3K/Akt/NF-kappaB signaling pathways [20]. Other work has also demonstrated that activation and clustering of the αMβ2 integrin in U937 cells and peripheral blood monocytes induced a translocation of PKCδ to the plasma membrane, whereby PKCδ is phosphorylated and thereby activated via the src family kinases Hck and Lyn to relay downstream signals involving the transcription factor Foxp1 [21]. The importance of PKCδ and associated MAP kinases during U937 and TUR cell differentiation and adherence has been demonstrated and suggested a functional relationship between these kinase systems and integrin-mediated attachment [22]. Indeed, previous work has documented the requirement of kinases including Src, PKCα, and PKCδ for integrin-mediated invasion by metastatic melanoma cells [23].

Other functional pathways which substantiate an involvement of MAP and src kinases in β_2 _integrin-relayed downstream signaling include the intracellular formation of reactive oxygen species which are properties of monocytes or activated macrophages. Thus, studies have demonstrated that inhibition of MAP kinases and the src kinase family blocked CD11b/CD18-mediated superoxide anion generation [24]. Thus, the enhanced expression of MAP kinase and pp60^src ^proteins in asCD11b cells as compared to pMTH1 control transfectants may elevate the TPA-induced superoxide anion production [25,26] due to the reduced availability of sufficient CD11b/CD18 protein complexes.

In summary, these findings suggested an important role of the functional β_2 _integrins to coordinate cell growth with signals for a monocytic maturation of U937 cells. Thus, a disturbed interaction with the cellular microenvironment by an abolished adherence can result in appropriate regulatory imbalances and subsequent apoptosis.

## Competing interests

The authors declare that they have no competing interests.

## Authors' contributions

AO contributed to Figure 1A and 1B and the quantification of Figure 2. KM contributed to Figure 4. GS contributed to Figure 1. RH designed the study, contributed to Figures 2, 3, 4 and 5 and drafted the manuscript. All authors have read and approved the final version of the manuscript.
